# Effects of hydrophilic coated catheters on urethral trauma, microtrauma and adverse events with intermittent catheterization in patients with bladder dysfunction: a systematic review and meta-analysis

**DOI:** 10.1007/s11255-022-03172-x

**Published:** 2022-04-21

**Authors:** Xi Liao, Yuwei Liu, Shiqi Liang, Ka Li

**Affiliations:** 1 West China School of Nursing, Sichuan University/West China Hospital, Sichuan University, Guoxue Xiang #37, Chengdu, 610041 Sichuan China; 2 West China Hospital, Sichuan University, Sichuan, 61004 China

**Keywords:** Hydrophilic catheters, Intermittent catheterization, Hematuria, Urethral stricture, Adverse events

## Abstract

**Background:**

Hydrophilic coated catheters are recommended to reduce the side effects of intermittent catheterization (IC) in patients with bladder dysfunction. However, there is lack of Level one evidence to support the use of this intervention.

**Search methods:**

Several electronic databases were systematically searched to evaluate complication incidences for hydrophilic coated (HC) and non-hydrophilic catheters (NHC).

**Results:**

Twelve studies were eligible for inclusion in the review. The meta-analyses exploring microscopic hematuria frequencies (RR = 0.69; 95% CI 0.52–0.90) and urethral stricture frequencies (RR = 0.28; 95% CI 0.13–0.60) showed a lower risk ratio associated with HC in comparison to NHC, whereas gross hematuria was no statistically significant difference in two groups. Subgroup analyses of gross hematuria which was grouped according to "catheterization frequency", "single/multiple catheterization" and "self/other catheterization” were performed and the values of combined RR were also no statistically significant difference.

**Conclusions:**

Compared with non-hydrophilic catheters, the hydrophilic coated catheters have positive significance in reducing the incidence of urethral microtrauma and the urethral stricture. However, more studies are warranted for evaluating effects of hydrophilic coated catheters on the incidence of gross hematuria.

**Supplementary Information:**

The online version contains supplementary material available at 10.1007/s11255-022-03172-x.

## Introduction

Causes of bladder dysfunction are neurogenic or non-neurogenic. Neurogenic bladder dysfunction is often secondary to spinal cord injury and central nervous system disease (multiple sclerosis or spina bifida), of which complications often manifest as urinary tract infections (UTI), urinary incontinence and upper urinary tract lesion [1]. Common non-neurogenic bladder dysfunction includes outlet obstruction, such as benign prostatic hyperplasia and postoperative urinary retention, which probably leads to vesicoureteral reflux. Bladder dysfunction hinders urine discharge, increases pressure in bladder, eventually causes urinary retention, which aggravates the risk of renal failure [2]. The treatment of bladder dysfunction is aimed at alleviating urinary incontinence, protecting the upper urinary tract, and improving bladder function as well as patients' quality of life.

Intermittent catheterization (IC) is a preferred treatment for patients with significant urination problems [3] which is used in 56% spinal cord injury patients for bladder management in the United States [4]. IC makes the bladder store a reasonable amount of urine at low pressure and empty it at appropriate intervals, which simulates physiological urinary function. Thereby, IC prevents overdistention and decreases pressure of bladder [5], improves blood circulation in bladder wall [6], reduces the incidence of urinary retention, and ultimately prevents deterioration of upper urinary tract [7].

However, there are non-negligible side effects of IC, such as inducible urethral trauma, microtrauma, urethral stricture, bladder stone and false passages formation [8–10]. In recent years, several types of conduits are gradually available for IC to solve these disadvantages, including especially gel pre-lubricated polyvinyl chloride (external lubricant at most) and hydrophilic-coated catheter (polyvinylpyrrolidone coated at most) [10]. Compared with gel pre-lubricated polyvinyl chloride, HC is increasingly used to reduce intubation friction, urethral injury and urethral adhesion due to its special hydrophilic lubrication characteristics and non-sensitization [11].

Three previously published meta-analyses investigated the effects of HC and non-hydrophilic catheters (NHC) on urethral bleeding morbidity in IC patients [3, 12, 13], however, the results were contradictory. In addition, these studies provide few reliable evidence of urethral microtrauma and urethral stricture which are also important outcomes in the early and late stages of IC, respectively, except for gross hematuria. Consequently, the aim of our study is to evaluate whether HC improves the direct adverse effects compared with NHC, especially in urethral trauma, microtrauma, urethral stricture and rare adverse events.

## Materials and methods

### Inclusion/exclusion criteria

*Population* Studies considering adults (over 18 years old), adolescents (12–18 years old) and children (less than 12 years old) population with bladder dysfunction requiring IC.

*Intervention* Hydrophilic catheters—single-use.

*Control* Non-hydrophilic catheters—single-use or multiple-use.

*Outcomes* Gross hematuria, urethral microtrauma (microscopic hematuria), urethral stricture, false passages, bladder stone.

*Study* Randomized controlled trials, controlled before-and-after study, prospective cohort studies and cross-over trials.

*Availability* English; full text.

### Data sources

We searched the following electronic databases to identify studies: Embase, PubMed, The Cochrane Central Register of Controlled Trials (CENTRAL), Web of Science, Medline, the Cumulative Index to Nursing and Allied Health Literature (CINAHL), British Nursing Index and three Chinese databases (The CNKI, Wan Fang Database and the VIP). The database has been established until December 31, 2021 and the search has been carried out by combining subject words with free words. English search terms include: 1. hydrophilic urethral catheters, hydrophilic-Coated Catheters, hydrophilic coated catheter. 2. Self-lubricated urethral catheters, pre-lubricated catheter, ultra-slippery, aqueous lubrication, surface wettability and lubrication, lubricant, aqueous lubrication, hydrogel coatings hydrogels, aqueous. 3. Reducing friction. 4. Urethra trauma, urethral micro trauma, urinary tract trauma, urethral epithelial micro-trauma. 5. Long-term follow-up study, long-term follow-up, reduce treatment-related complications, adverse events, false passages, urethral stricture, bladder stone. At the same time, the references of the included literatures have been manually retrieved to supplement the relevant literatures.

### Literature screening

Two evaluators read the obtained literature independently. After excluding the trials that clearly did not meet the inclusion criteria, the full text of the trials that might meet the inclusion criteria was read to determine whether they really met the inclusion criteria. After the cross-check, if there is a disagreement, a third party will assist in adjudication. Data extraction was performed using standardized forms of the Cochrane Collaboration. The extracted contents include: ① basic information of the included study, ② baseline characteristics included in the study, ③ specific details of the intervention including catheter material/catheter brand, the coating type and the lubrication mode, ④ key factors for the risk of bias include catheter size, self-catheterization or other-catheterization, single-use or multiple-use of catheterization, daily frequency of intubation, ⑤ Outcome indicators and outcome measures.

### Bias risk assessment for included studies

Methodologic quality was independently assessed by 2 reviewers using Cochrane.

### Statistical analysis

Risk Ratios (RRs) were used as a measure of the relationship between hydrophilic or non-hydrophilic catheters and outcome indicators. The 95% confidence interval (CI) for the dichotomous data was calculated. The pooled RRs were adopted the Mantel–Haenszel method. If there were no events in one or both arms, the Peto method was used. The percentage of variability of each study attributable to heterogeneity beyond chance was evaluated by the chi-square test (*P* < 0.10) and *I*^2^ statistics. According to heterogeneity test, we adopted the random effects model (*I*^*2*^ > 50%, *P* < 0.10) or the fixed effects model. Then, the probability of publication bias was evaluated with Egger’s test and funnel plots. All statistical analyses were conducted with Stata15.0.

## Results

### Literature screening process and results

**Fig. 1:**
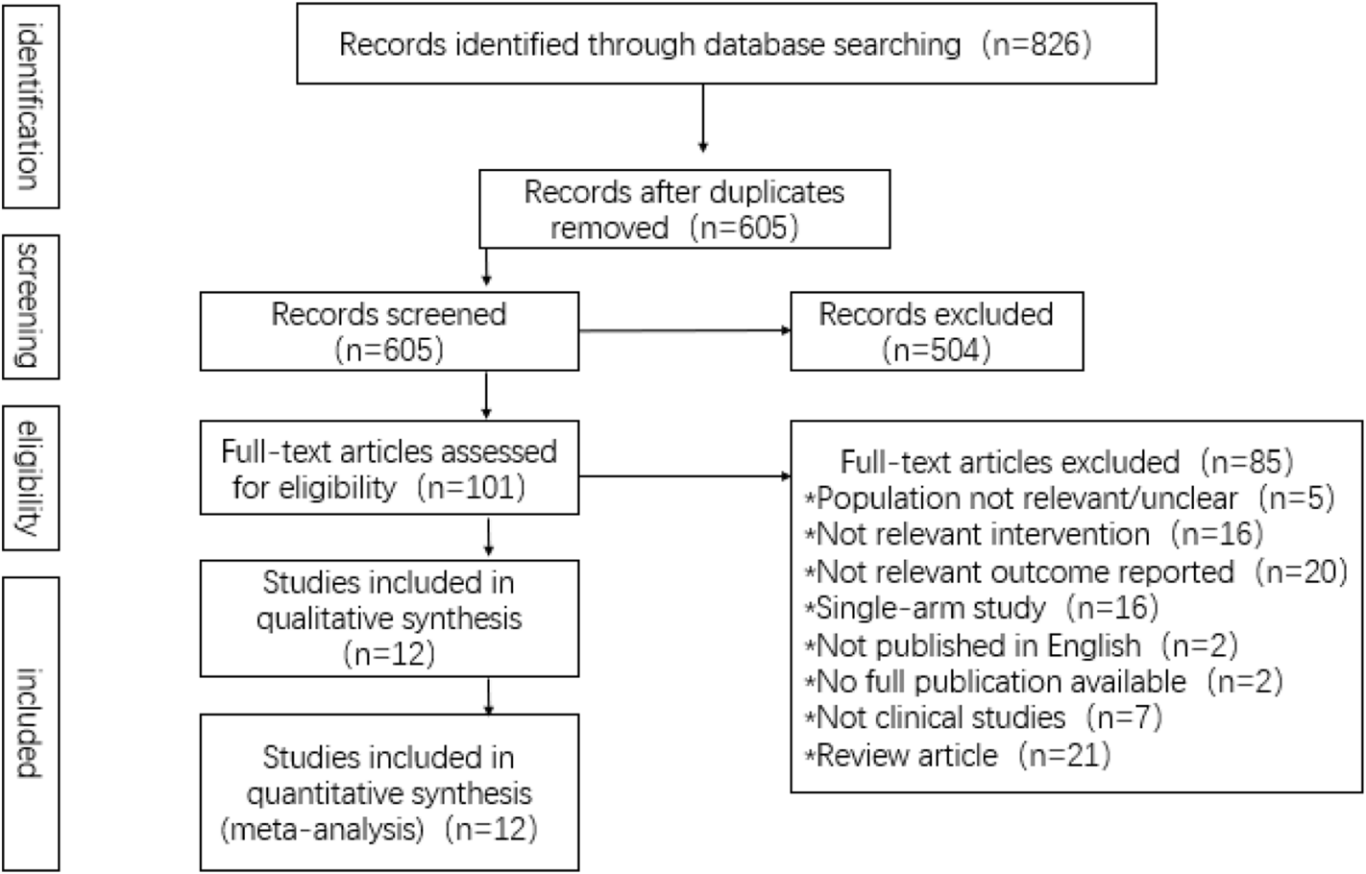
PRISMA flow diagram—clinical search strategy

Figure 1 shows the selection process at each step and the reasons for excluded studies. Finally, 12 papers containing 850 participants met the inclusion criteria [14–25], including 9 randomized controlled trials [14, 15, 17, 19, 21–23, 25], 1 controlled before-and-after study [20], 1 prospective cohort studies [18], and 1 cross-over trials [16]. Table 1 illustrated patients’ characteristics (age and gender), catheter materials and catheter size. Meta-regression was performed with the year of publication, male proportion and age as independent variables, and the results showed that the regression equation had no statistical significance (*p* > 0.05).

**Table 1:** Summary of extracted clinical data

Study	Location	Age	Gender ^a^	Catheter material (brand), C/T^b^	Size
William DeFoor (2017) [14]	America	12.9/13.6	38/40	C: unknown T: unknown/lofric (Wellspect Healthcare)	Unknown
De Ridder (2005) [15]	Spain, Belgium	37.5±14.6/ 36.7±14.6	M	C:PVC^c^ (Conveen,Coloplast) T:PU^d^/Speedicath (Coloplast)	ch10,12,14
Pachler (1999) [16]	Denmark	71.3	M	C:PVC (Mentor santa barbara) T:PVC/lofric (Astra pharmaceuticals)	Unknown
Diana D. Cardenas (2011) [17]	America, Canada	35.1±13.2/ 37.2±14.4	100/39	C:PVC (Conen)T: PU/Speedicath	Unknown
Tariq Burki (2019) [18]	Saudi Arabia	5	47/54	C:PE^e^ T: unknown	Unknown
Ronald (1996)	America	11.7±3.8/ 12.1±5.7	M	C:PVC (Mentor) T:unknown/Lofric	11.5 ± 2.5/11.1 ± 2.1
Wyndaele (2000) [20]	Belgium	45±15	M	C:unknown T:unknown/Urocath-Gel1	12–14 French
Luca Cindolo (2003) [21]	Italy	62.3/67.4	80/20	C:PVC T:PVC/EasiCath (Coloplast, Denmark)	12-Charr
Sataa Sallami (2010) [22]	Tunisia	62/60.9	M	C:PVC T:unknown/LoFric (Astra Tech; Molndal, Sweden)	Unknown/Number 16 or 18
Jonathan et al. (2003) [23]	America	39.8±12.9/ 39.6±16	M	C:PVC T: unknown/Lofric	MOST are 14Fr, a few are 16Fr, 12Fr
Stensballe (2005) [24]	Denmark	24	M	C:silica gel or PVC (incare1 advance plus, Hollister inc,USA) T:unknown/speedicath (Conveen, ColoplastA/S Denmark)	CH12
Kjaergaard (1994) [25]	Denmark	68	M	C:no T: unknown/LoFric	Unknown

^a^Gender: M/ F (male/ female)

^b^T: hydrophilic coated (HC); C: non-hydrophilic catheters (NHC)

^c^PVC: polyvinyl chloride

^d^PU: polyurethane

^e^PE: polyethylene

### Risk bias assessment form for included studies

**Fig. 2:**
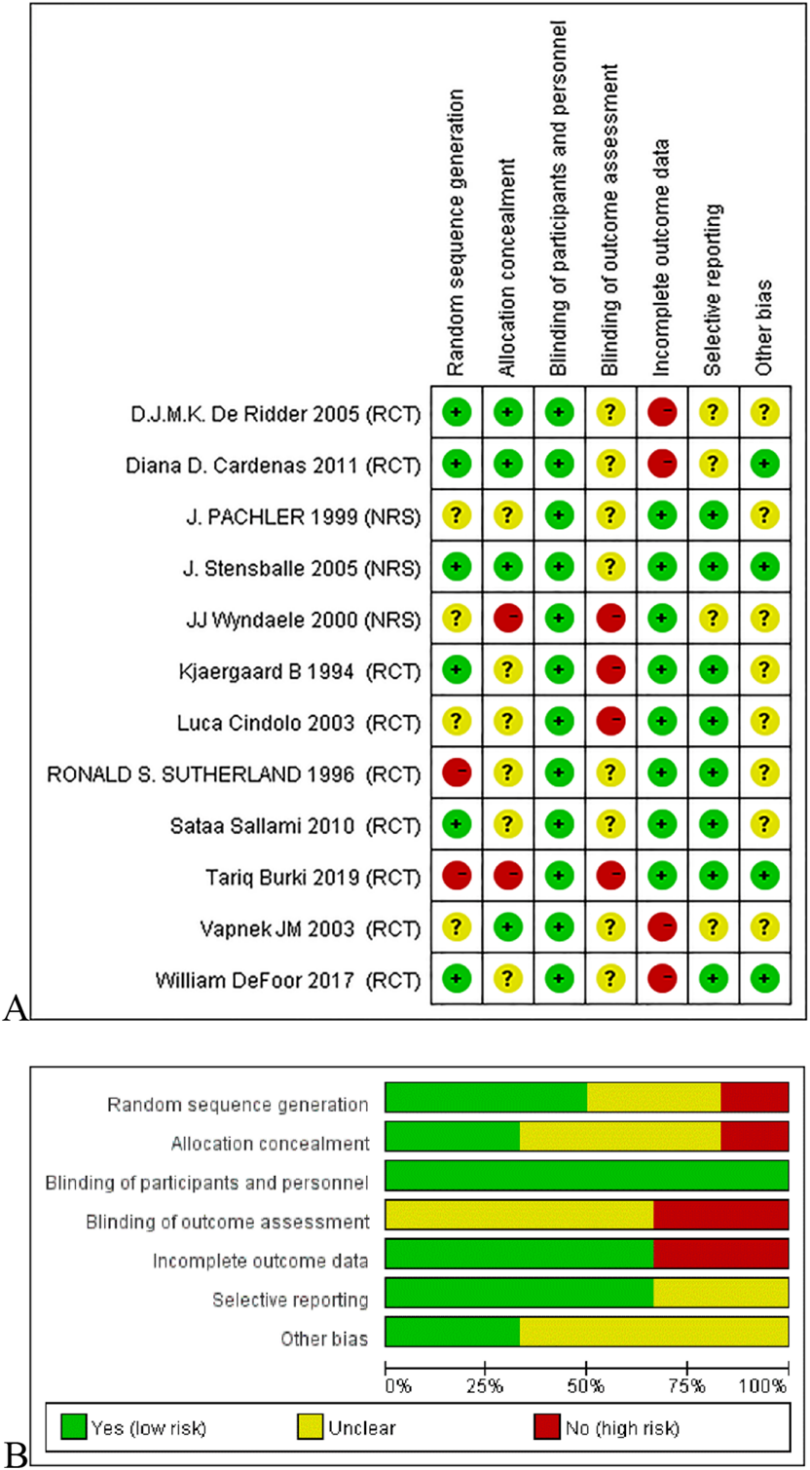
**A** Risk of bias summary for RCT (*n* = 9) and NRS (*n* = 3); **B** risk of bias graph for all included studies (*n* = 12). *RCT* randomized controlled trials, *NRS* non-randomized controlled trials

In these studies, blinding of participants and interveners were not possible, but even unblinded methods were considered unlikely to have an impact on objective evaluation indicators. Therefore, they were classified as low risk. Patient withdrawal (an average of 17.71%) was common in the literature [14–17, 19, 22–25], which was an unbalanced and potentially biased factors (Fig. 2).

### The results of the study

#### Gross hematuria

**Fig. 3:**
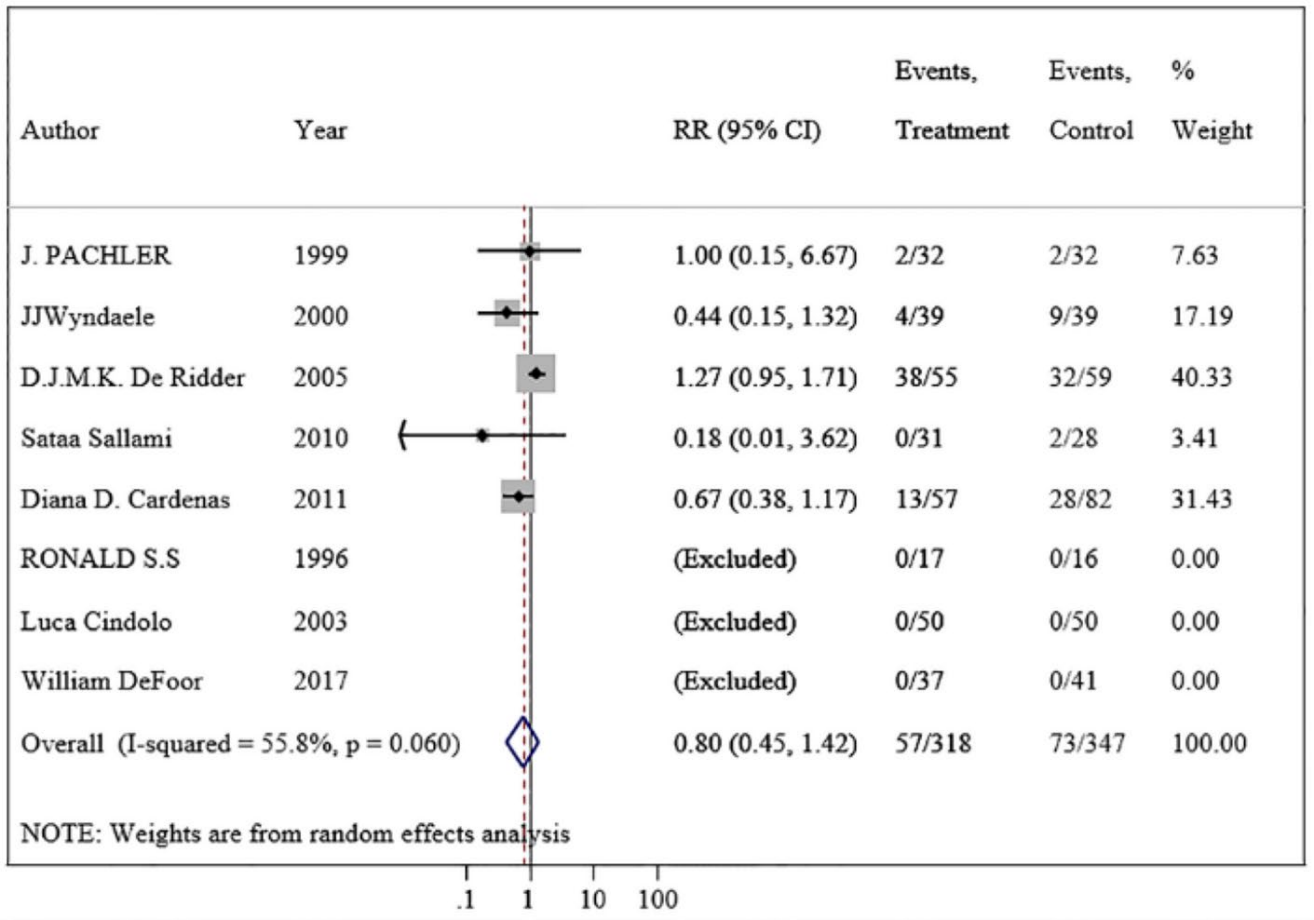
Meta-analysis comparing hydrophilic catheter with non-hydrophilic catheter, evaluating gross hematuria

**Fig. 4:**
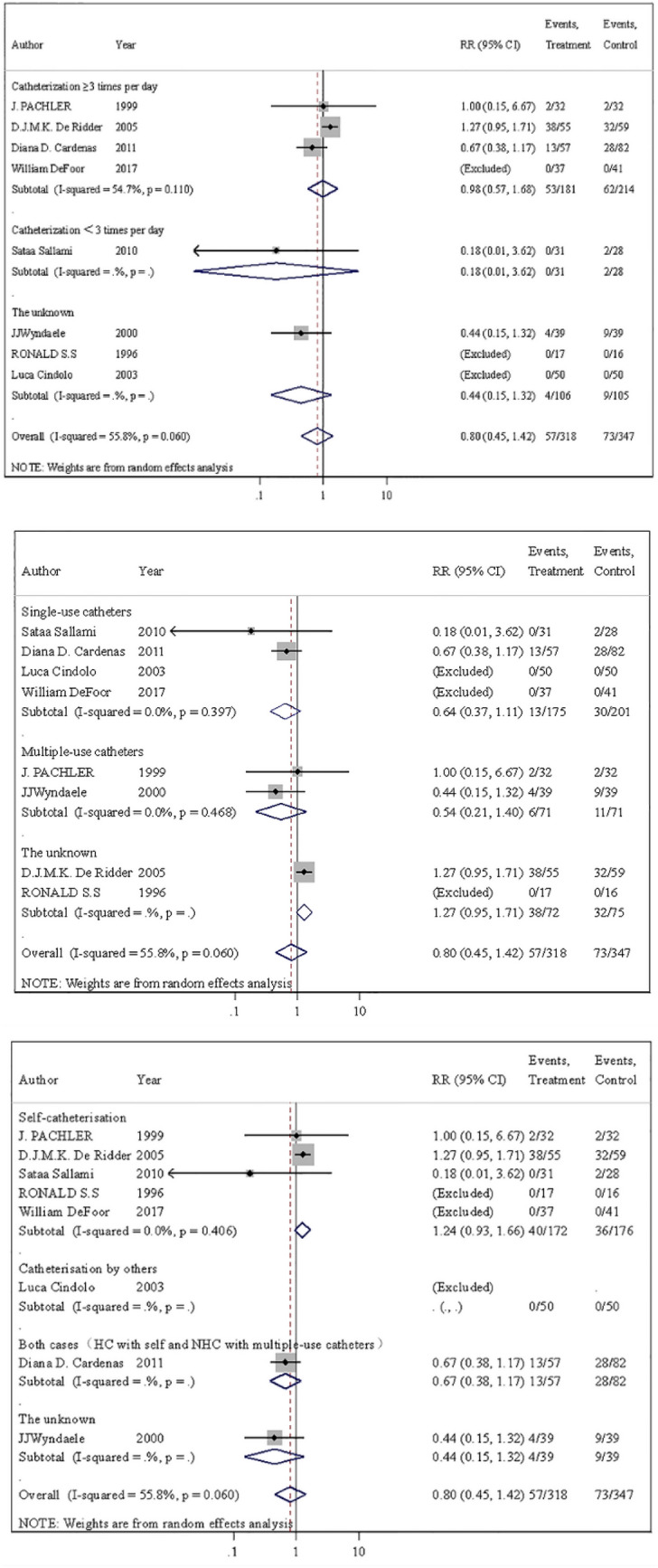
Meta-analysis comparing hydrophilic catheter with non-hydrophilic catheter, evaluating subgroup analysis of gross hematuria

Studies have used different terms such as urethral bleeding, hematuria and gross hematuria to describe the same condition. A total of eight trials reported the number of patients with gross hematuria [14–17, 19–22]. The incidence of gross hematuria was 17.9% (57/318) in patients using hydrophilic catheters and 21.0% (73/347) in patients using non-hydrophilic catheters (RR = 0.80; 95% CI 0.45–1.42) (Fig. 3). The risk of gross hematuria was not statistically significant between two groups. As "catheterization frequency", "single/multiple catheterization" and "self/other catheterization" are key indicators for gross hematuria incidence, we performed subgroup analysis for the three aspects. Figure 4 shows that there was still no statistically significant difference in the risk of gross hematuria incidence. In addition, the proportion of male was found that it did not affect the results of the final forest plot of gross hematuria by meta-regression (additional Fig. 3④). Moreover, there was also no evidence of heterogeneity (*p* = 0.060; *I*^2^ = 55.8%) or publication bias (*t* = − 1.94, *P* = 0.148) (additional Fig. 3②). For the results of the sensitivity analysis, all the included studies were within the confidence interval except one study at the lower limit of the 95% CI (additional Fig. 3③). In brief, HC did not significantly improve the incidence of gross hematuria compared with NHC.

#### Microscopic hematuria

**Fig. 5:**
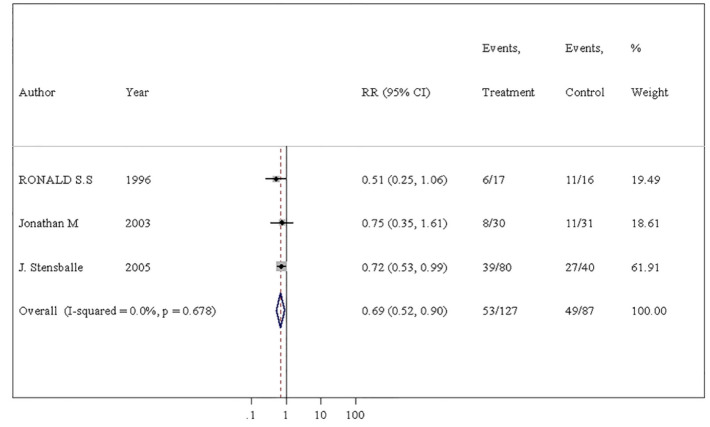
Meta-analysis comparing hydrophilic catheter with non-hydrophilic catheter, evaluating microscopic hematuria

In this study, we considered microscopic hematuria as the following definition: the presence of red blood cells (RBC) in high power field under the microscope. There were 3 trials in 12 studies for microscopic hematuria in our study [19, 23, 24]. The incidence of microscopic hematuria was 41.7% (53/127) in patients using hydrophilic catheters and 56.3% (49/87) in patients using non-hydrophilic catheters (RR = 0.69; 95% CI, 0.52–0.90) (Fig. 5). The difference between two groups was statistically significant, indicating that the risk of microscopic hematuria with hydrophilic catheters was only 69% of that in non-hydrophilic group. There was also no evidence of heterogeneity (*p* = 0.678; *I*^2^ = 0.0%) or publication bias (*t* = − 0.65, *P* = 0.633) (additional Fig. 5②). For the results of the sensitivity analysis, the included studies were all within the CI (additional Fig. 5③). In short, HC significantly improved the incidence of microscopic hematuria compared with NHC.

#### Urethral stricture

**Fig. 6:**
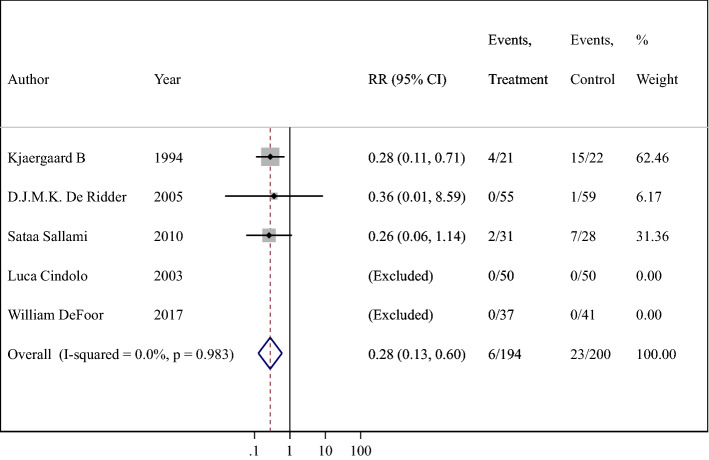
Meta-analysis comparing hydrophilic catheter with non-hydrophilic catheter, evaluating urethral stricture

The method for stricture evaluation is maximum flow rate < 14 mL/s or endoscopic or radiographic examination. A total of five trials reported the number of patients with urethral stricture [14, 15, 21, 22, 25]. The incidence of urethral stricture was 3.1% (6/194) in patients using hydrophilic catheters and 11.5% (23/200) in patients using non-hydrophilic catheters (RR = 0.28; 95% CI 0.13–0.60) (Fig. 6). The difference between two groups was statistically significant, suggesting that the risk of urethral stricture with hydrophilic catheters was only 28% of that in the non-hydrophilic group. There was also no evidence of heterogeneity (*P* = 0.983; *I*^2^ = 0.0%) or publication bias (*t* = 0.69, *P* = 0.617) (additional Fig. 6②). Five studies were all within the 95% CI about the sensitivity analysis (additional Fig. 6③). In a word, HC significantly improved the incidence of urethral stricture compared with NHC.

#### Rare adverse events

In addition to hematuria and urethral stricture, false passages and bladder stone are also rare adverse reactions after intubation in patients with bladder dysfunction. There were two studies focusing on the incidence of false passages [14, 20] and another two studies on bladder stone morbidity [18, 23]. Wyndaele [20] enrolled 39 patients who had been using NHC for IC over a number of years and switched to urocath-gel hydrophilic lubricated catheter for 1 month. It was found that only NHC group had one false passage. William [14] included children with neurogenic bladder dysfunction and divided them into 41 patients with NHC and 37 patients with HC. There were no false passages patients found in both groups. Jonathan [23] included 30 patients with HC and 31 patients with NHC for neurogenic bladder dysfunction, and found that one patient in each group had bladder stone. Tariq [18] included 101 children with spina bifida and divided them into HC and NHC groups. There were no bladder stones in the two groups. The incidences of both indicators were low after IC, and there was no difference between the two groups.

## Discussion

Since Dr. Lapides proposed that using of IC as an alternative way to urinary diversion in《Urology》in 1972 [6], IC has become the globally recognized standard for the treatment of neurogenic bladder dysfunction and has been usually used in managements for various urinary system disease [26]. Generally, IC improves the quality of patients’ life through removing long-standing drainage tubes and drainage bags [2]. Initially, catheters for IC were mainly made of latex and rubber. However, these catheters were gradually taken placed by polyvinyl chloride (PVC) catheters due to their sensitization, hardness and difficulty in catheterization [27]. In addition, the practice of re-using catheters with same tube in IC has changed over the past 10 years, for example most patients with intermittent self-catheterization (ISC) were required to use disposable catheters during catheterization [2].

Under the guidance of healthcare workers, almost all patients with bladder dysfunction could get benefits from IC [2]. IC changes the pattern of urinary management in patients with bladder dysfunction because of its various advantages. In addition to decreasing mortality caused by kidney deterioration [28], IC also reduces the harmful effects of long-term indwelling urinary catheters, including urinary tract infections (UTIs) [29], traumatic hypospadias, urinary fistula and even bladder cancer [30]. However, there are still unavoidable complications including mechanical stimulation and mucosa injuries for IC, such as pain and urethral injury. Applying external lubricant is a traditional method to reduce mucosa friction and adhesion during catheterization. Common external lubricants cover Vaseline, paraffin oil, gel, lidocaine cream, amiodarone and ketamine [31]. Nevertheless, the application of external lubricant on the surface of urinary duct has plentiful limitations such as uneven application, cumbersome operation, weak lubrication effect and short residence time. In addition, anesthetic lubricant such as lidocaine cream contains additives that cause allergic reactions [32].

In recent years, water lubrication, which is an ideal solution to ultralow friction of medical catheter has received growing attentions. Hydrophilic coated catheters are usually made of PVC material and polyvinylpyrrolidone coated (PVP coated). PVP is a polymer with hydrophilic groups [33]. After the PVP hydrophilic groups are combined with a lubricating fluid (such as water or saline), the interface between the surface of catheter and the urethral mucosa forms a smooth area composed mainly of water molecules [24]. Direct contact between the surfaces is avoided during sliding process, thus greatly reducing friction coefficient and mucosal injury [24, 34, 35]. Furthermore, PVP coated possibly reduce a potential risk of urethral stricture caused by repeatedly intubation [22, 36]. Meanwhile, PVP coated is able to reduce the adsorption of fibrinogen and fibronectin, as well as the deposition of hydroxyapatite on the tube surface [34], potentially resulting in lower incidence of bladder stone.

Generally, gross hematuria is used as an indicator to estimate urethral trauma. However, the results of previous researches were contradictory in regard to whether gross hematuria could be reduced by HC [3, 12, 13]. Two meta-analyses concluded that HC was associated with a reduced risk of urethral bleeding compared with NHC [12, 13], but another research suggested a higher risk of hematuria in the HC group [3]. Simultaneously, the results from the three meta-analyses were challenged due to their inclusion, heterogeneity and bias risk analysis.

Gross hematuria is a more serious outcome indicator, so it is not a favorable indicator for reflecting the early condition of urethral damage. Innovatively, our study assessed urethral microtrauma using microscopic hematuria. Except for urethral bleeding, there are few studies evaluating whether HC reduce the incidence of adverse events, such as urethral stricture, false passages and bladder stone. In our study, HC made positive contributions to reducing the incidence of urethral microtrauma and urethral stricture compared with NHC, whereas gross hematuria was no significant difference. More studies are needed to further confirm the association between HC and these indicators in the future.

### Implications for clinical practice

Due to the limitations of the study population and relevant intervention measures, the results of previous studies were contradictory and difficult to be generalized. Our study included a broad population of men and women of all ages with IC. There were no strict restrictions on the influencing factors, including catheterization frequency, self-catheterization or other-catheterization, single-use or multiple-use and the intubation environment. Therefore, our results regarding the complications of HC have broad adaptability to guide clinical practice.

### Call for future studies

More high-quality, large-scale RCT studies are urgently needed. Recommendations for future research are as follows: ① The inclusion and exclusion criteria of study subjects should be clarified; ② The specific details of the intervention should be clarified including catheter material/catheter brand, the coating type and the way of lubrication; ③ key factors for the risk of bias need to be controlled including catheter size, total duration of intubation, time to start catheterization, self-catheterization or other catheterization, single-use or multiple-use of catheterization and catheterization frequency; ④ Call for clear definition of outcome indicators and specification of outcome measures.

### Limitations

Our study still had some aspects for improving: ① Due to the wide heterogeneity of study subjects, study design, outcome measurement methods, as well as the small number of included literatures, it was difficult to conduct meta-subgroup analysis about long term adverse events such as urethral stricture. Therefore, we only performed subgroup analysis for gross hematuria; ② Risk of bias covers “little blinding of participants and interveners” and “the differences in patient drop-off between the two groups”, which perhaps impact study results; ③ The majority of our data was in males and it would be a non-negligible influence factor for IC. However, the objects are only men in the current literature which was eligible for inclusion in these two indicators of microscopic hematuria and urethral stricture.

## Conclusion

This meta-analysis supports the benefits of using hydrophilic coated catheters for IC in patients with bladder dysfunction, including reduced incidence of microscopic hematuria and urethral stricture. However, whether HC reduces the risk of gross hematuria has not been proven. While waiting for more evidence, it is recommended to select a more appropriate catheter type of IC combined safety, efficacy, cost effectiveness and patient satisfaction. Patients are advised to use hydrophilic coated catheter as the first treatment option when the condition permits to reduce urethral complications and offers higher comfort [20]. In this study, we evaluated the effects of HC and NHC on urethral trauma, microtrauma, urethral stricture and rare adverse events, demonstrating that HC is a better intubation method for patients with bladder dysfunction.

## Supplementary Information

Below is the link to the electronic supplementary material.

Gross Hematuria: the proportion of male was found that it did not affect the results of the final forest plot of gross hematuria by meta-regression (TIF 194 kb)

Gross Hematuria: there was also no evidence of heterogeneity (p=0.060; I-squared =55.8%) or publication bias (t=-1.94, P=0.148) (TIF 127 kb)

Gross Hematuria: For the results of the sensitivity analysis, all the included studies were within the confidence interval except one study at the lower limit of the 95% CI (TIF 223 kb)

Microscopic hematuria: There was also no evidence of heterogeneity (p=0.678;I-squared =0.0%) or publication bias (t=-0.65, P=0.633) (TIF 128 kb)

Microscopic hematuria: For the results of the sensitivity analysis, the included studies were all within the CI (TIF 161 kb)

Urethral stricture: There was also no evidence of heterogeneity (p=0.983;I-squared =0.0%) or publication bias (t=0.69, P=0.617) (TIF 135 kb)

Urethral stricture: Five studies were all within the 95% CI about the sensitivity analysis (TIF 191 kb)
