# Supplementary figures and images for: Effects of hydrophilic coated catheters on urethral trauma, microtrauma and adverse events with intermittent catheterization in patients with bladder dysfunction: a systematic review and meta-analysis
Source: Int Urol Nephrol. 2022 Apr 21;54(7):1461–70. doi: 10.1007/s11255-022-03172-x (PMC9184422; doi:10.1007/s11255-022-03172-x)

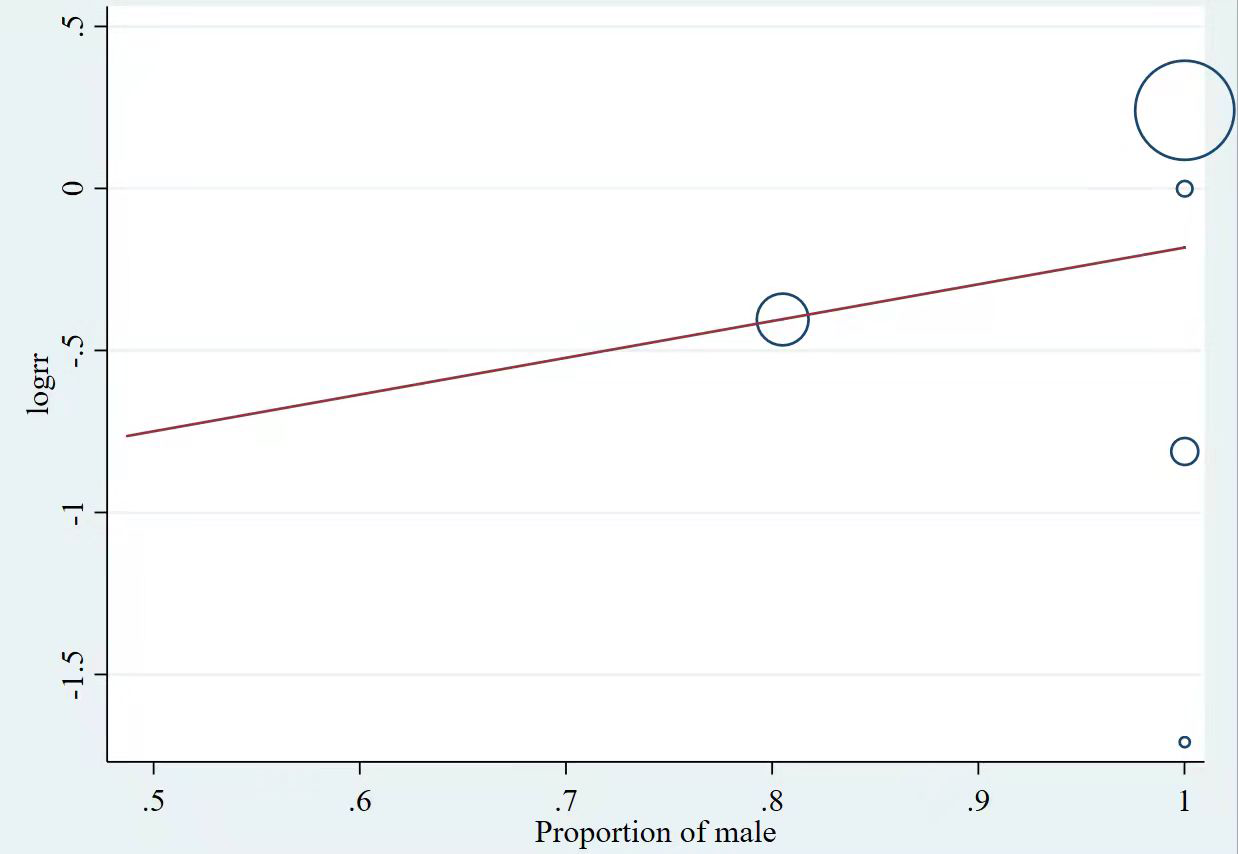

Supplement: Supplementary file 1 — Gross Hematuria: the proportion of male was found that it did not affect the results of the final forest plot of gross hematuria by meta-regression (TIF 194 kb) [file 11255_2022_3172_MOESM1_ESM.tif]

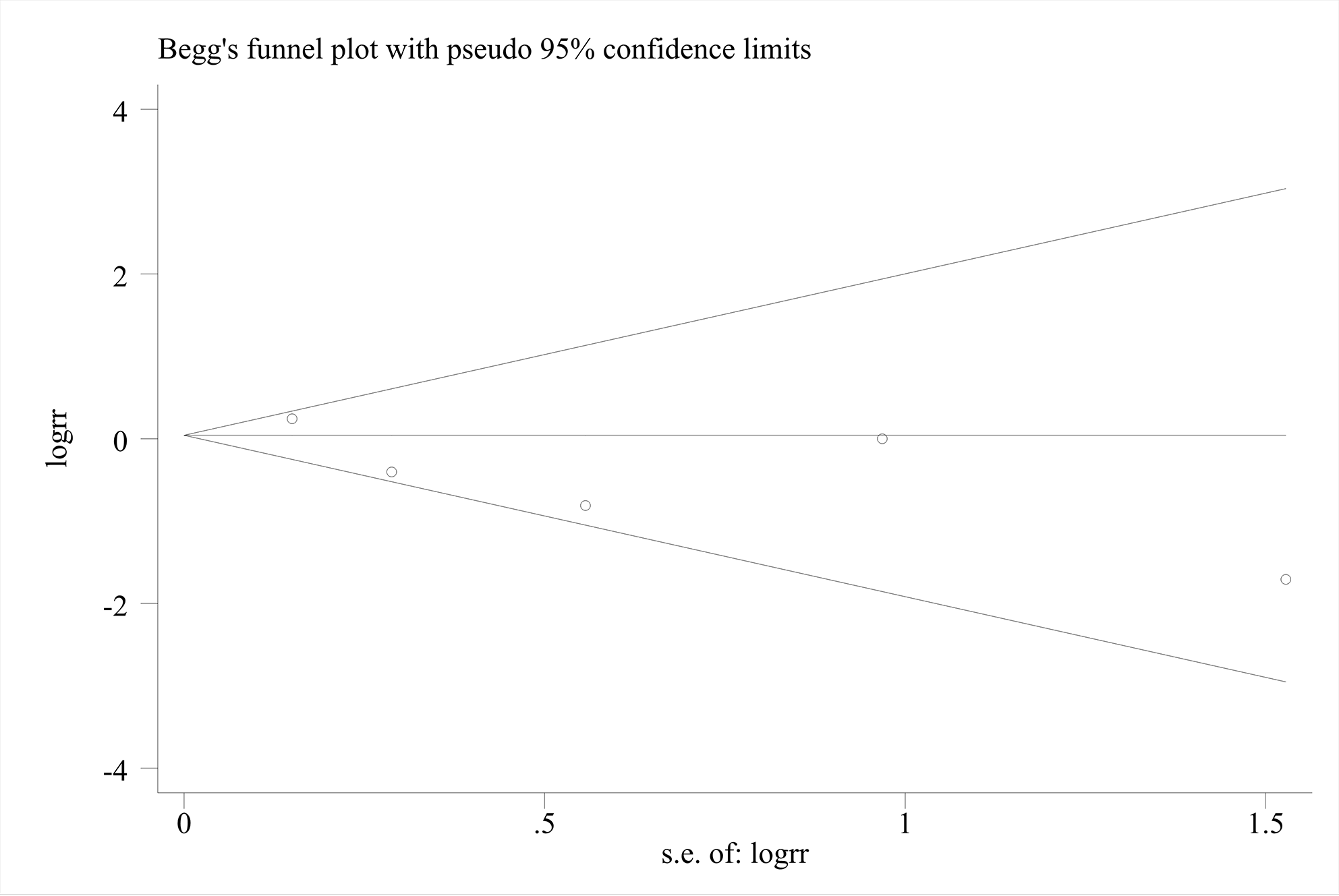

Supplement: Supplementary file 2 — Gross Hematuria: there was also no evidence of heterogeneity (p=0.060; I-squared =55.8%) or publication bias (t=-1.94, P=0.148) (TIF 127 kb) [file 11255_2022_3172_MOESM2_ESM.tif]

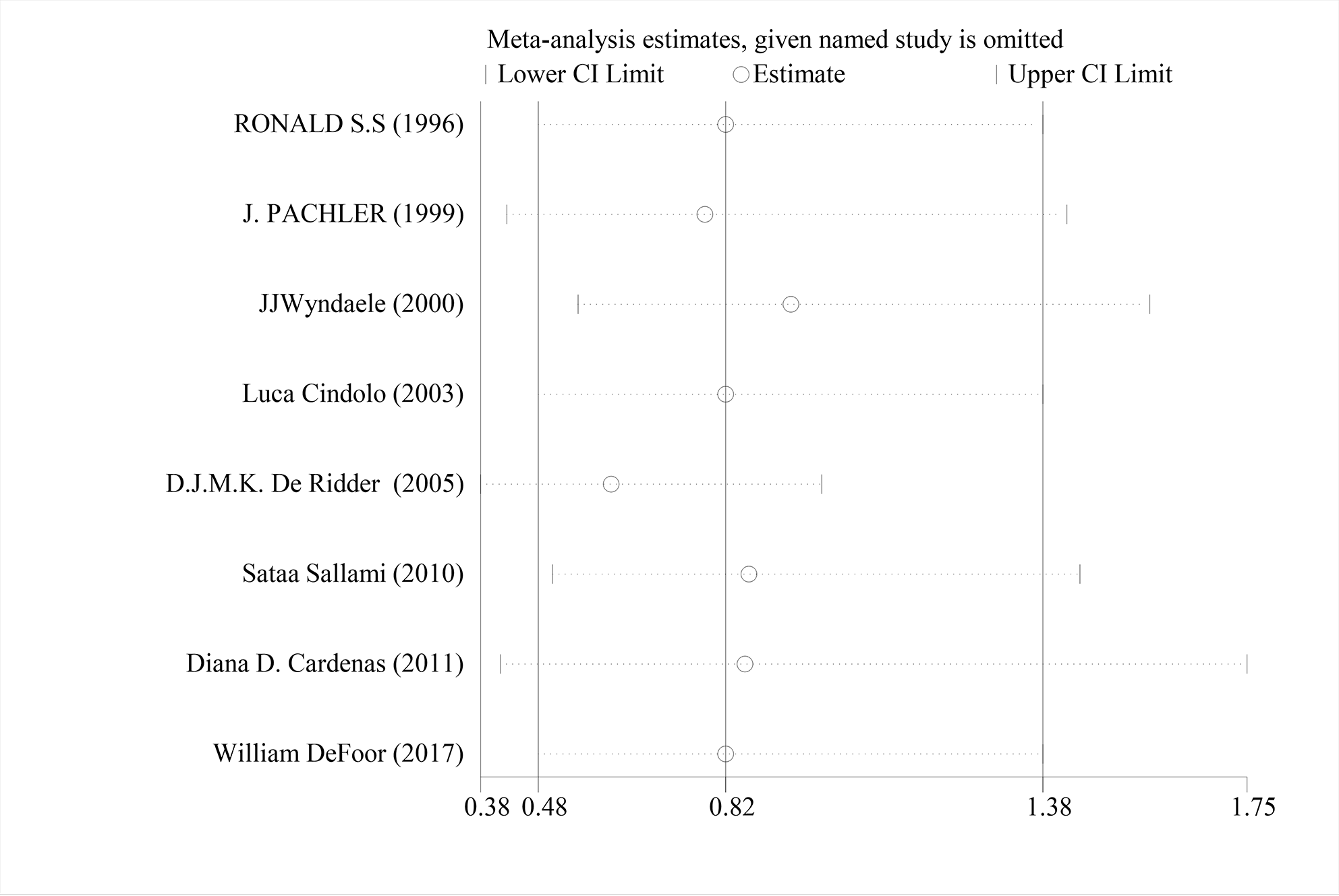

Supplement: Supplementary file 3 — Gross Hematuria: For the results of the sensitivity analysis, all the included studies were within the confidence interval except one study at the lower limit of the 95% CI (TIF 223 kb) [file 11255_2022_3172_MOESM3_ESM.tif]

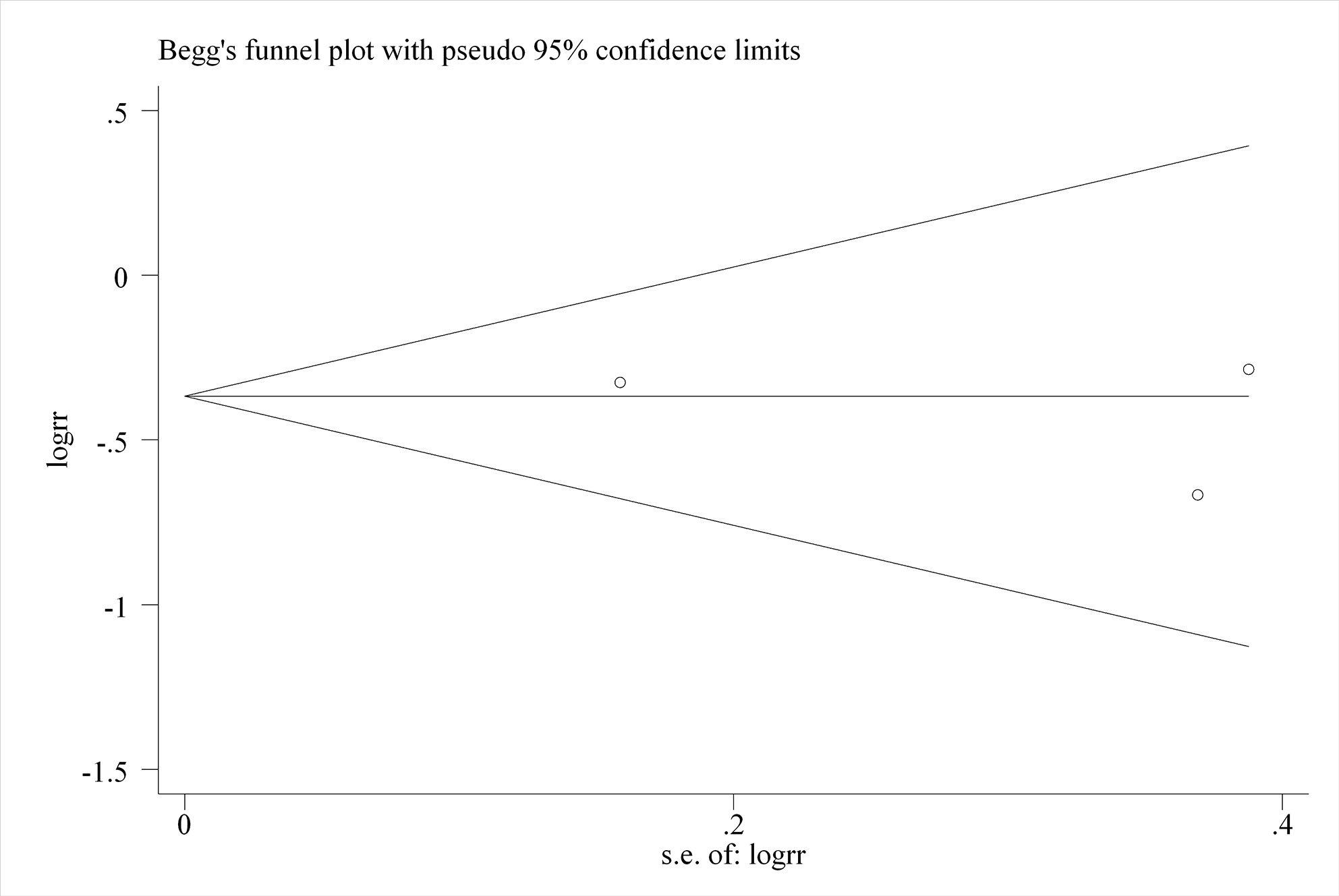

Supplement: Supplementary file 4 — Microscopic hematuria: There was also no evidence of heterogeneity (p=0.678;I-squared =0.0%) or publication bias (t=-0.65, P=0.633) (TIF 128 kb) [file 11255_2022_3172_MOESM4_ESM.tif]

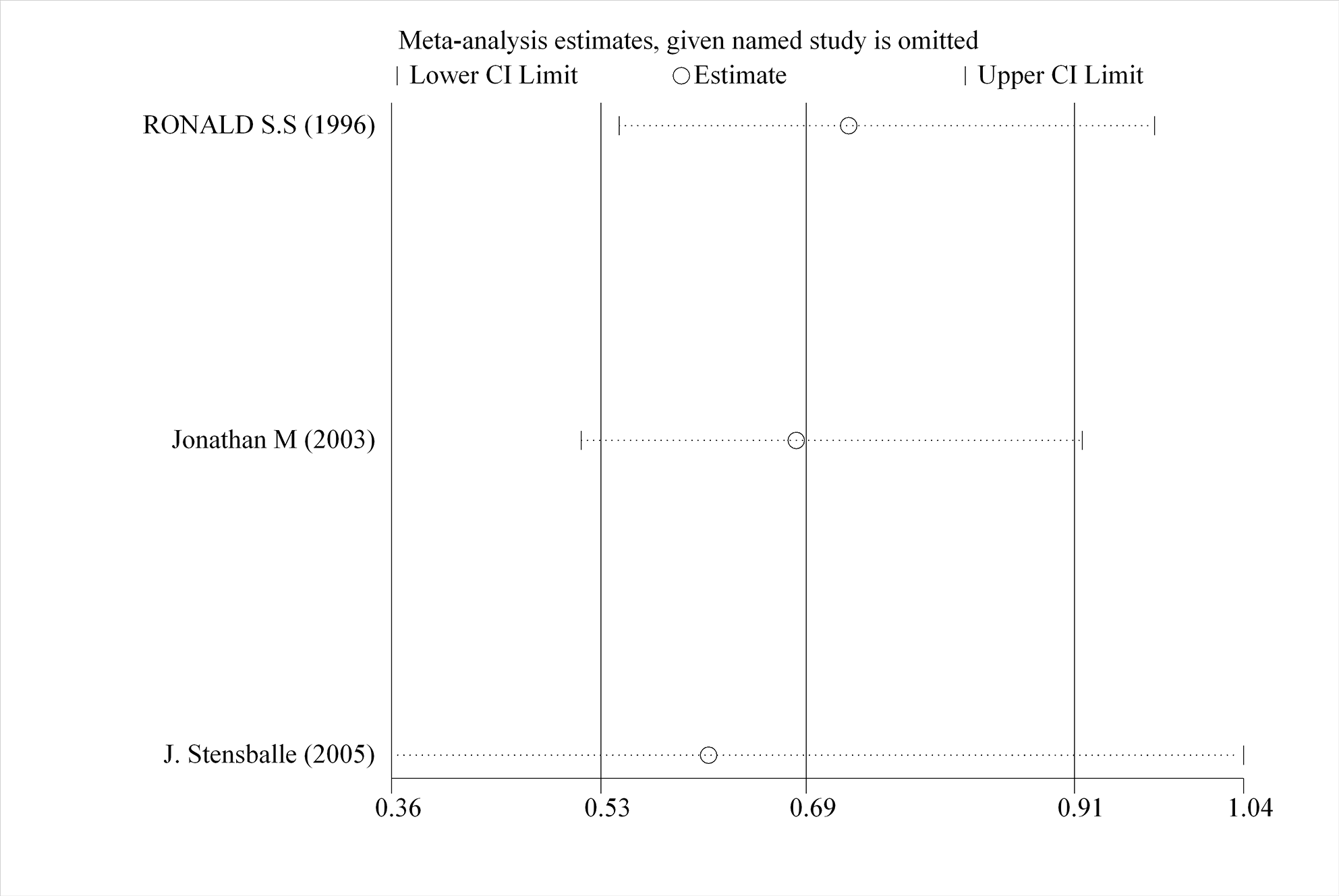

Supplement: Supplementary file 5 — Microscopic hematuria: For the results of the sensitivity analysis, the included studies were all within the CI (TIF 161 kb) [file 11255_2022_3172_MOESM5_ESM.tif]

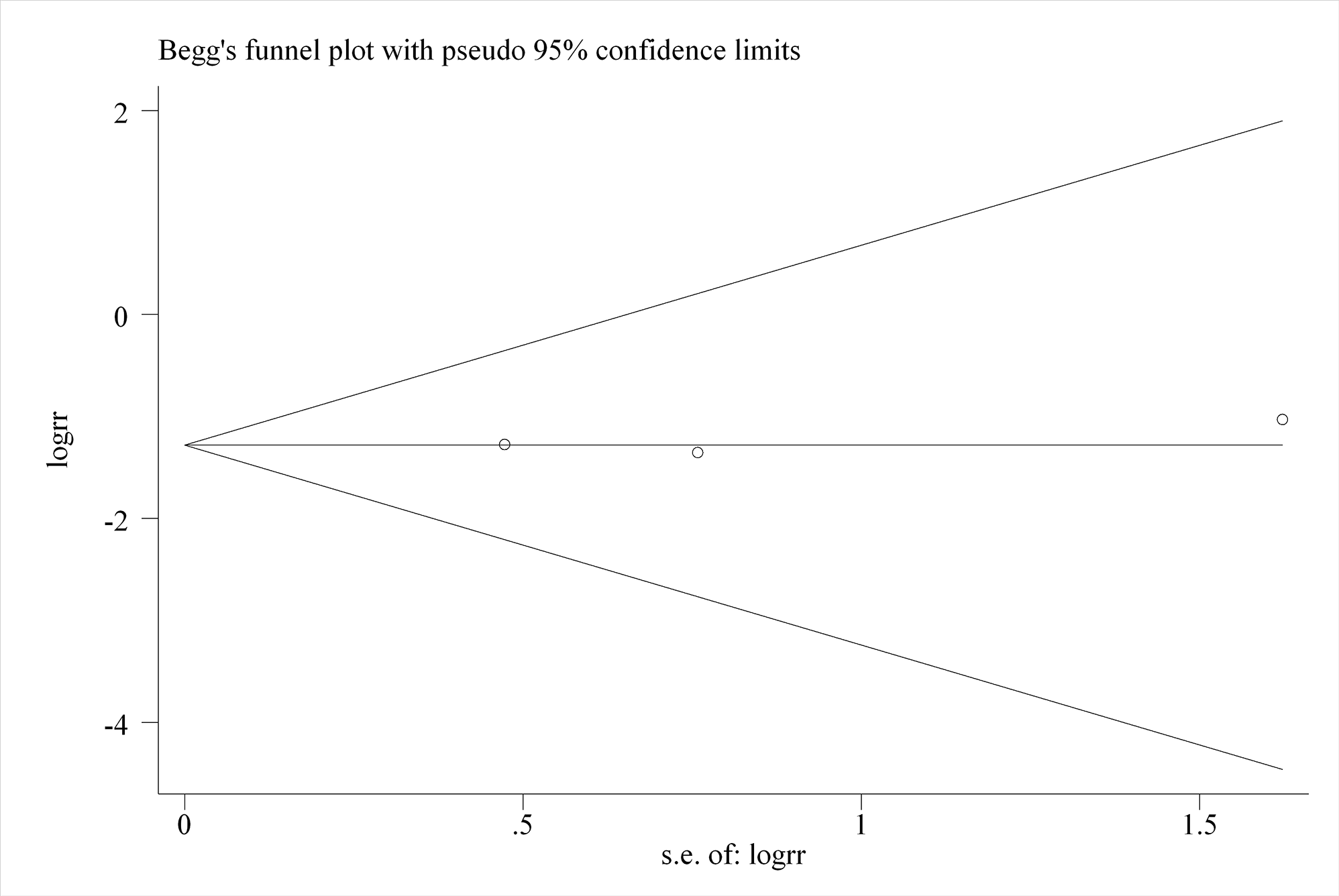

Supplement: Supplementary file 6 — Urethral stricture: There was also no evidence of heterogeneity (p=0.983;I-squared =0.0%) or publication bias (t=0.69, P=0.617) (TIF 135 kb) [file 11255_2022_3172_MOESM6_ESM.tif]

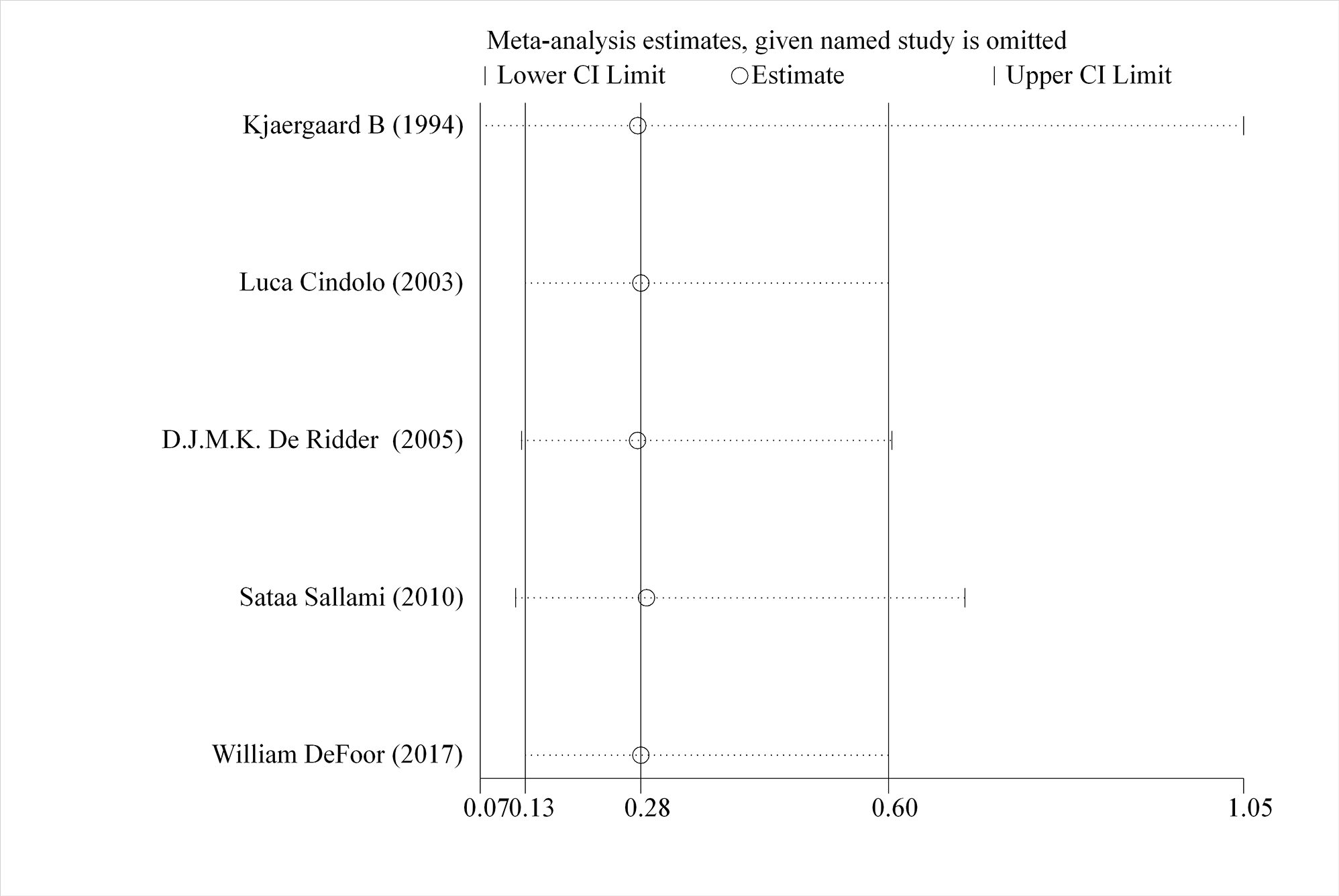

Supplement: Supplementary file 7 — Urethral stricture: Five studies were all within the 95% CI about the sensitivity analysis (TIF 191 kb) [file 11255_2022_3172_MOESM7_ESM.tif]
